# Neuropsychological differences between frontotemporal lobar degeneration and Alzheimer’s disease

**DOI:** 10.1590/S1980-57642009DN20300011

**Published:** 2008

**Authors:** Claudia Sellitto Porto, Valeria Santoro Bahia, Sonia Maria Dozzi Brucki, Paulo Caramelli, Ricardo Nitrini

**Affiliations:** 1 PhD. Behavioral and Cognitive Neurology Unit, Department of Neurology of the University of São Paulo School of Medicine and Cognitive Disorders Reference Center (CEREDIC), Hospital das Clínicas of the University of São Paulo School of Medicine, São Paulo (SP), Brazil.; 2 MD, PhD. Behavioral and Cognitive Neurology Unit, Department of Neurology of the University of São Paulo School of Medicine and Cognitive Disorders Reference Center (CEREDIC), Hospital das Clínicas of the University of São Paulo School of Medicine, São Paulo (SP), Brazil.; 3 MD, PhD. Behavioral and Cognitive Neurology Unit, Department of Internal Medicine, Faculty of Medicine, Federal University of Minas Gerais, Belo Horizonte (MG), Brazil.

**Keywords:** neuropsychological assessment, memory, executive functions, Alzheimer disease, frontotemporal lobar degeneration

## Abstract

Memory impairment is the main clinical feature in Alzheimer disease (AD), whereas in frontotemporal lobar degeneration (FTLD) behavioral and language disorders predominate.

**Objectives:**

To investigate possible differences between the neuropsychological performance in FTLD and AD.

**Methods:**

Fifty-six AD patients (mean age=72.98±7.43; mean schooling=9.62±4.68; 35 women and 21 men), 17 FTLD patients (mean age=67.64±7.93; mean schooling=12.12±4.77; 9 women and 8 men), and 60 controls (mean age=68.90±7.48; mean schooling=10.72±4.74; 42 women and 18 men) were submitted to a Dementia Rating Scale (DRS) and a comprehensive neuropsychological evaluation composed of tasks assessing attention, visuoperceptual abilities, constructive abilities, executive functions, memory and language.

**Results:**

DRS total score and subscales were not able to differentiate FTLD from AD patients. However, FTLD and AD patients showed statistically significant differences in performance in tests of verbal (Logical Memory, Rey Auditory Verbal Learning Test) and visual (Visual Reproduction, recall of the Rey Complex Figure) episodic memory, verbal immediate memory (Logical Memory), attention with interference (Trail Making Test – Part B), verbal fluency (semantic and phonemic) and concept formation (WCST).

**Conclusion:**

Contrary to expectations, only a few tasks executive function tasks (Trail Making Test – Part B, F.A.S. and WCST) and two memory tests (verbal and visual episodic memory tests) were able to differentiate between FTLD and AD patients.

## Abstract (pt)

Comprometimento de memória é a principal característica clínica na doença de Alzheimer (DA), enquanto na degeneração lobar fronto-temporal (DLFT) alterações de linguagem e comportamentais são predominantes.

**Objetivos:**

Investigar possíveis diferenças entre o desempenho neuropsicológico de pacientes com DLFT e pacientes com DA.

**Métodos:**

56 pacientes com DA (média de idade=72,98±7,43; média de escolaridade=9,62±4,68; 35 mulheres e 21 homens), 17 pacientes com DLFT (média de idade=67,64±7,93; média de escolaridade=12,12±4,77; 9 mulheres e 8 homens) e 60 controles (média de idade=68,90±7,48; média de escolaridade=10,72±4,74; 42 mulheres e 18 homens) foram submetidos à Escala de Avaliação de Demência (Dementia Rating Scale – DRS) e à extensa avaliação neuropsicológica composta de tarefas que examinam atenção, habilidades visuais-perceptuais, habilidades construtivas, funções executivas, memória e linguagem.

**Resultados:**

O escore total e as subescalas da DRS não foram capazes de diferenciar pacientes com DA de DLFT. Pacientes com DLFT e DA demonstraram diferenças estatisticamente significativa em testes de memória episódica verbal (Memória Lógica, Teste de Aprendizagem Verbal Auditiva de Rey) e visual (Reprodução Visual e evocação da Figura Complexa de Rey), memória imediata verbal (Memória Lógica), atenção com interferência (Trail Making Test – Parte B) e fluência verbal (semântica e fonêmica).

**Conclusão:**

Ao contrário do esperado, somente algumas tarefas de função executiva (Trail Making Test – Parte B, F.A.S. e WCST) e dois testes de memória (testes de memória episódica verbal e visual) foram capazes de diferenciar pacientes com DLFT de pacientes com DA.

Memory impairment is the most prominent deficit in Alzheimer disease (AD). A more heterogeneous pattern of cognitive impairment, however, is seen in frontotemporal lobar degeneration (FTLD), a neurodegenerative disorder characterized by progressive behavioral and/or language disorders or semantic memory changes.^1^ Neary et al.^1^ distinguished three variants of FTLD: the frontal variant of frontotemporal dementia (FTD), semantic dementia (SD) and progressive non-fluent aphasia (PNFA). In FTD, behavioral symptoms are predominant, while oral production and semantic deficits are observed in PNFA and SD, respectively.

Clinical differentiation between FTLD and AD remains a great challenge, especially in the clinical setting. Mendez et al.^2^ demonstrated that neuropsychological evaluation did not distinguish frontotemporal dementia (FTD) from other causes of dementia while some studies advocate the use of behavioral scales over neuropsychological tests to differentiate\ FTD from AD patients.^3^

In a recent study, Liscic et al.^4^ investigated clinical and psychometric differences between neuropathogically confirmed FTLD (without or with concomitant AD pathological features) and AD, finding that behavioral and language features were good discriminators between the two conditions. However, FTLD patients or their relatives can also report memory loss complaints, although – in most cases – this is related to attention and working memory deficits.

The main objective of this study was to investigate possible differences between the performance of patients with FTLD and AD on neuropsychological tests.

## Methods

The study involved 73 patients (44 women and 29 men), aged 50 to 84 years (mean=71.73±7.83), with schooling ranging from 3 to 17 years (mean=10.21±4.79), attended by members of the Behavioral and Cognitive Neurology Unit of the Department of Neurology of the University of São Paulo School of Medicine, in Brazil. All patients were submitted to appropriate laboratory tests and to structural neuroimaging (computed tomography (CT) or magnetic resonance (MR) of the skull), the Mini-Mental State Examination (MMSE)^5,6^ and the Brief Cognitive Screening Battery (BCSB).^7^ Information on performance in daily life activities was obtained through the Pfeffer Functional Activities Questionnaire,^8^ which was applied to an informant.

The probable AD group was composed of 56 individuals, aged 54 to 84 years (mean=72.98±7.43), with schooling ranging from 3 to 17 years (mean=9.62±4.68), comprising 35 women and 21 men. The clinical diagnosis of mild dementia was based on the Diagnostic and Statistical Manual of Mental Disorders, Third Edition, revised (DSM-III-R) criteria;^9^ whereas the diagnosis of probable AD was based on the National Institute of Neurological Disorders and Communicative Disorders and Stroke-Alzheimer’s Disease and Related Disorders Association (NINCDS-ADRDA) criteria.^10^

The FTLD group was composed of 17 patients (SD= 3; PNFA=4; FTD=10), aged 50 to 80 years (mean 67.64±7.93), with schooling ranging from 4 to 16 years (mean=12.12±4.77), 9 women and 8 men. The diagnosis of FTLD was based on the criteria of Neary et al.^1^

The control group (60 subjects; mean age=68.90±7.48; mean schooling=10.72±4.74; 42 women and 18 men) was composed of spouses or consorts of the patients, or volunteers from the community, with no memory disorders and who were fully independent in terms of daily living activities. Subjects with neurological disease, history of alcoholism, depression, or any other psychiatric disorder, non-corrected visual or auditory disorders, motor disorders, or users of psychotropic drugs that could affect cognitive functions were excluded. Chronic diseases such as arterial hypertension, diabetes mellitus and cardiac disorders, if adequately controlled, were not criteria for exclusion. All controls were submitted to the MMSE, BCSB and Memory Complaint Questionnaire (MAC-Q)^11^ or to the Informant Questionnaire on Cognitive Decline in the Elderly (IQCODE),^12,13^ administered to an informant.

Patients and controls were submitted to the Dementia Rating Scale^14-16^ and to a comprehensive neuropsychological evaluation, which included the following tests: visual and verbal memory tests (Visual Reproduction subtest of the Wechsler Memory Scale – Revised (WMS-R),^17^ Rey Complex Figure – delayed recall,^18^ Logical Memory subtest (WMS-R),^17^ Rey Auditory Verbal Learning Test (RAVLT)^19^), constructive abilities (Block Design subtest – Wechsler Adult Intelligence Scale (WAIS),^20^ Rey Complex Figure copy^18^), visual perception (Hooper Visual Organization Test^21^ and Raven’s Progressive Matrices^22^), language (Boston Naming Test)^23^), and executive functions (Trail Making Test versions A and B,^24^ Stroop Test,^24^ Wisconsin Card Sorting Test (WCST)^24^ and phonemic verbal fluency (F.A.S.)^24^).

The study was approved by the Research and Ethics Committee of Hospital das Clínicas from the University of São Paulo School of Medicine. All subjects who agreed to participate signed a written informed consent.

## Statistical analysis

In order to evaluate associations between the categorical variables and the results, the Pearson Chi-Squared test was performed. When the variables were continuous, the comparisons were made for two samples by the Mann-Whitney test, and for more than two, by the Kruskall-Wallis test.

Alpha risk was considered to be less than or equal to 5% for type 1 error, and beta risk greater than or equal to 20% for type II error.

All statistical analysis was carried out using the Statistical Package for the Social Sciences (SPSS) program, version 10.0.

## Results

No differences related to schooling (p=0.105) or gender (p=0.394) were found between control and patient groups, but a statistically significant difference related to age was observed (p=0.004).

There was also a significant difference between the performance of both patient groups and controls on the DRS, in total score (p<0.001) and all subscales. No differences between AD and FTLD patients were seen in total score (p=0.881) or on the DRS subscales.

Regarding the comparison between AD and FTLD patients, there were statistically significant differences in visual and verbal episodic memory tasks, in verbal fluency, as well as in tests evaluating attention and executive functions (Table 1).

**Table 1:** Performance on neuropsychological tests in AD and FTLD patients.

Neuropsychological assessment	mean±SD AD	mean±SD FTLD	P
Hooper	63.63±19.92	49.28±26.41	0.105
Block Design (WAIS)	7.38±3.38	8.29±4.85	0.563
Rey Figure - copy	21.35±9.69	22.17±11.75	0.568
Rey Figure - memory	0.73±2.73	3.35±5.02	0.016
Trail Making - Part A	92.27±46.22	95.80±87.58	0.198
Trail Making - Part B	223.57±78.91	159.83±80.36	0.015
Logical Memory (WMS-R) - immediate	13.87±8.96	20.00 v 8.49	0.013
Logical Memory (WMS-R) -30'	1.39±3.01	6.81±7.37	0.001
Visual Reproduction (WMS-R) - immediate	14.30±7.07	19.50±11.02	0.069
Visual Reproduction (WMS-R) - 30'	0.69±2.94	5.87±9.68	0.007
RAVLT - total	21.49±7.73	22.31±11.27	0.918
RAVLT - 30'	1.11±3.03	3.23±3.21	0.005
WCST	0.46±0.63	1.50±1.30	0.024
Raven's Colored Matrices	17.50±7.12	14.50±17.67	0.854
BNT	34.67±9.09	40.66±11.20	0.155
Verbal fluency - supermarket	13.41±4.97	10.47±4.50	0.032
Verbal fluency - FAS	25.84±10.41	16.55±13.24	0.029

SD, standard deviation; AD, Alzheimer disease; FTLD, frontotemporal lobar degeneration; WAIS, Wechsler Adult Intelligence Scale; WMS-R, Wechsler Memory Scale - Revised; RAVLT, Rey Auditory Verbal Learning Test; WCST, Wisconsin Card Sorting Test; BNT, Boston Naming Test.

AD patients showed worse performance than FTLD patients in immediate recall on the Logical Memory test (p=0.013) and on the Trail Making Test – Part B (p=0.015).

## Discussion

Memory impairment is the hallmark feature of AD while in FTLD episodic memory remains relatively preserved,^25,26^ that could explain the results of our study demonstrating that verbal and visual episodic memory tests were able to differentiate between the two patient groups. Wicklund et al.^26^ compared AD patients, frontal variant of FTLD patients, and controls on two memory tests: story memory and word list recall. The results demonstrated patients with frontal variant of FTLD recalled more information from the story and more words after a delay than AD patients. Heidler-Gary et al.^27^ also demonstrated that AD was characterized by severe impairment in verbal learning, delayed recall and that two variants of FTLD (FTD and PNFA) were characterized by relatively normal scores on verbal learning and recall.

AD and FTLD patients performed differently in immediate story recall (the Logical Memory subtest of WMS-R), with greater impairment shown by AD patients. Wicklund et al.^26^ found that individuals with the behavioral variant of FTLD were able to immediately recall more information from the story than AD patients. Studies^28,29^ have shown low performance in verbal short- term memory tasks in AD. This deficit is attributed to problems in attention, coordination and integration processes stemming from impaired executive control processes. Lezak^30^ reported that immediate story recall remained stable in middle age and declined progressively thereafter. In our study age differences between groups may have influenced the results.

Alescio-Lautier et al.^31^ affirmed that certain attentional mechanisms are impaired early in AD. Patients with AD showed greater impairment on the divided attention test, evaluated through the Trail Making Test – Part B, than FTLD and controls. Belleville et al.^32^ demonstrated that mild AD patients presented severe impairment in divided attention, manipulation capacities and inhibition.

Only two executive function tasks, both of which assess executive function, were able to differentiate AD from FTDL patients: phonemic verbal fluency and WCST. In the study by Liscic et al.,^4^ the FTLD group performed significantly worse on word fluency than the AD group.

In our study, the DRS, using either the total or subscale scores, was not effective in discriminating between AD and FTLD patients, suggesting that this scale is not useful to differentiate these two groups. This finding was somewhat unexpected because the subscales of the DRS evaluate specific items of cognition such as Initiation/Perseveration (I/P) that are usually more disturbed in FTLD or memory, which is more involved in AD. On the I/P subscale, the semantic verbal fluency test accounted for 75% of the total score of this subscale. Verbal fluency impairment is associated to initial stages of AD and also to FTLD.^25,33^ The tasks of the Memory subscale proved to easy or poor to differentiate between AD and FTLD groups.

In this study, verbal and visual episodic memory tests were better discriminators of the two groups whereas comprehensive neuropsychological evaluation was unable to clearly distinguish AD from FTLD individuals.

## Figures and Tables

**Figure 1:**
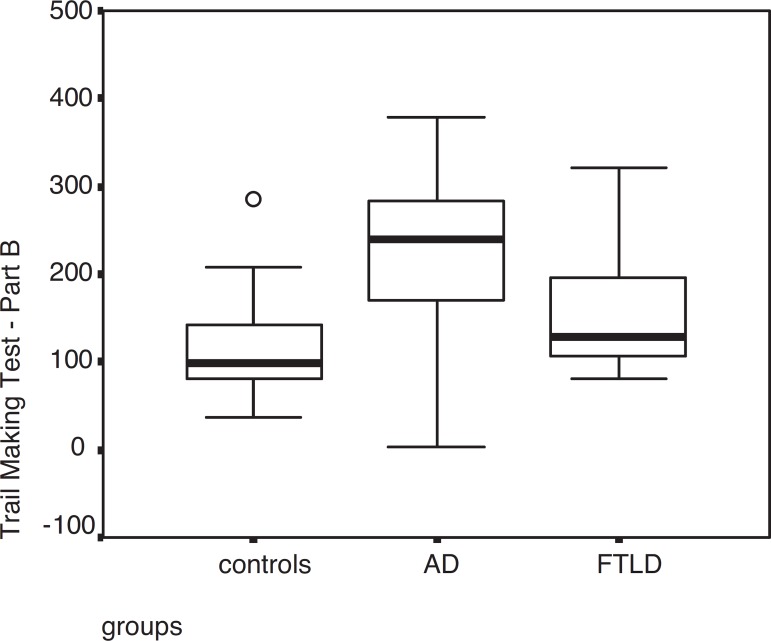
Performance among AD, FTLD patients and controls on the Trail Making Test – Part B.

**Figure 2:**
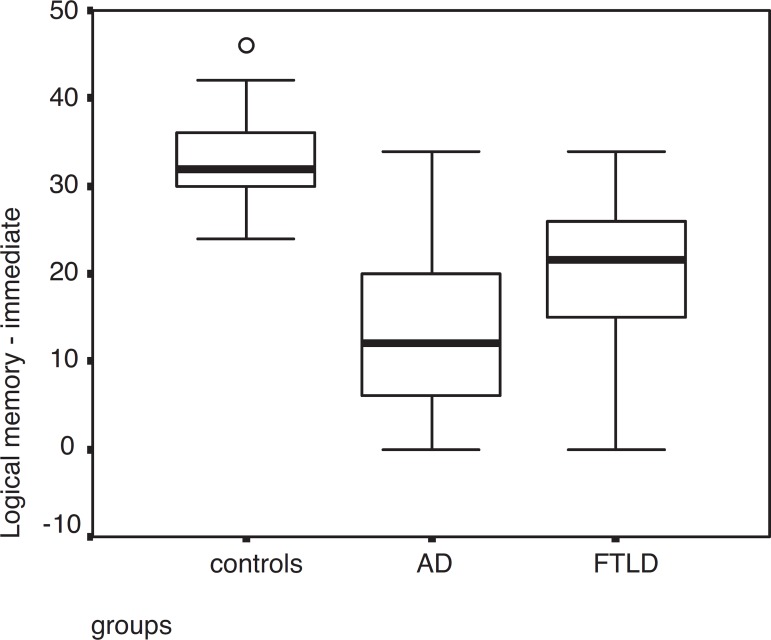
Performance among AD, FTLD patients and controls in the Logical Memory test – immediate.
